# Screening of 50,539 newborns for congenital hypothyroidism: optimization of TSH cut-off values and seasonal impact in clinical practice

**DOI:** 10.3389/fendo.2025.1616748

**Published:** 2025-06-27

**Authors:** Lixian Zhang, Kun Lin, Xinrong Han

**Affiliations:** ^1^ Neonatal Disease Screening Department, Putian Maternal and Child Health Care Hospital, The Affiliated Hospital (Group) of Putian University, Putian, China; ^2^ Department of Genetic and Prenatal Diagnosis Center, The Affiliated Hospital (Group) of Putian University, Putian, China

**Keywords:** newborn disease screening, congenital hypothyroidism, thyroid-stimulating hormone (TSH), cut-off optimization, seasonal impact

## Abstract

**Objective:**

To analyze the incidence of congenital hypothyroidism (CH) in Putian, optimize the thyroid-stimulating hormone (TSH) screening cut-off value, and improve diagnostic efficiency and accuracy.

**Methods:**

A retrospective analysis was conducted on TSH screening data from 50,539 newborns in Putian between July 2020 and November 2022. TSH concentrations in dried blood spots were measured using time-resolved fluorescence immunoassay (TRFIA). The optimal cut-off value was evaluated using percentile analysis and receiver operating characteristic (ROC) curves. Confirmatory tests included serum TSH, free thyroxine (FT4), and thyroid ultrasound.

**Results:**

The detection rate of CH was 1:1,232 (41 cases), with an overall detection rate of 1:555 (including 50 cases of hyperthyrotropinemia). The P99 percentile method determined a TSH cut-off of 11.1 μIU/mL, while ROC curve analysis indicated an optimal cut-off range of 9.33–9.43 μIU/mL (sensitivity 100%, specificity 98.1%, area under the curve [AUC] = 0.997, *P* < 0.0001). Adopting a revised cut-off of 9.5 μIU/mL reduced recall rates by 10.62% but missed 1 case of hyperthyrotropinemia. Initial TSH positivity exhibited seasonal fluctuations, with higher rates in winter than summer. Among 68 initially negative cases with subsequent elevated venous TSH, 2 were confirmed as CH, highlighting the need for secondary screening in high-risk groups.

**Conclusion:**

A TSH cut-off of 9.5 μIU/mL optimizes CH screening in Putian, balancing sensitivity (100%) and specificity (98.1%). Seasonal TSH variations and high-risk cases (e.g., preterm infants) highlight the need for tailored protocols. This approach improves screening efficiency and reduces recalls, supporting region-specific adjustments.

## 1 Introduction

Congenital hypothyroidism (CH) is one of the most common congenital endocrine disorders in newborns, primarily caused by thyroid dysgenesis or defects in hormone synthesis. If not promptly diagnosed and treated, it can lead to irreversible neurological damage in children, such as intellectual disability and growth retardation (1). Although European consensus guidelines indicate that early screening and standardized treatment enable most CH patients to achieve normal neurodevelopment (2), the clinical manifestations of CH during the neonatal period are often atypical, making it prone to underdiagnosis. Thus, universal newborn screening is critical for preventing disabling complications (3).

In China, neonatal CH screening predominantly relies on thyroid-stimulating hormone (TSH) testing using dried blood spots. However, significant regional variations exist in TSH cut-off values (reported domestically as 4.82–18.0 μIU/mL), which are closely associated with detection methodologies, population characteristics, and environmental factors (4). Blind adoption of uniform cut-offs may increase false-positive rates (adding psychological burden to families) or result in false-negative cases (delaying treatment).

Recent studies suggest that optimizing TSH cut-offs requires balancing sensitivity and specificity based on regional epidemiological data (5). Additionally, external factors such as ambient temperature may influence physiological TSH fluctuations, leading to seasonal variations in screening outcomes (6). Nevertheless, large-scale studies on TSH cut-off optimization remain scarce in southeastern coastal regions of China. This study, based on screening data from 50,539 newborns, proposes an optimized TSH cut-off for Putian using percentile analysis and ROC curve methodology, while also exploring the impact of seasonal changes on screening results. The aim is to provide a scientific foundation for regionally tailored and precise neonatal CH screening strategies.

## 2 Materials and methods

### 2.1 Research object

This study included 50,539 newborns (27,317 males and 23,222 females) born in Putian, Fujian Province, who underwent neonatal disease screening between July 2020 and November 2022. Inclusion criteria: Heel blood collection completed within 72 hours of birth, with guardians signing informed consent for neonatal genetic metabolic disease screening. Exclusion criteria: a) Neonates transferred to other hospitals or deceased due to critical illnesses without completing the full screening protocol; b) Inadequate feeding; c) Neonates who received blood transfusions; d) Cases where legal guardians (either parent) voluntarily withdrew from the study. Ethical approval was obtained from the Medical Ethics Committee of Putian University Affiliated Hospital Medical Group (No: 202415).

## 3 Research methods

### 3.1 Specimen collection and processing

Heel blood samples were collected by trained professionals 72 hours after birth (ensuring adequate feeding) following the Technical Specifications for Neonatal Disease Screening (2010 Edition). Blood was drawn from the medial or lateral heel and applied to specialized filter paper (The model of the filter paper is Scheicher and Schuell 903#) to form uniform blood spots with a diameter ≥8 mm (four spots per sample). The filter paper was air-dried on a ventilated rack, sealed, stored at 2–8°C, and transported twice weekly via cold chain to the Putian Maternal and Child Health Hospital Neonatal Screening Center. Exclusion criteria: a) Qualified dried blood spots cannot be provided:Insufficient blood volume (<8 mm diameter)、Visible contamination、Non-homogeneous blood penetration、Overlapping blood drops; b) Neonates transferred to other hospitals or deceased due to critical illnesses without completing the full screening protocol; c) Inadequate feeding; d) Neonates who received blood transfusions; e) Cases where legal guardians (either parent) voluntarily withdrew from the study.

### 3.2 TSH initial inspection measurement

The TSH concentration in dried blood spots was measured using a PerkinElmer GSP fully automated fluorescence immunoassay analyzer (Model 2021-0010) with matching reagent kits, employing time-resolved fluorescence immunoassay (TRFIA). Quality Control: Each batch included duplicate high- and low-value control samples, with results complying with Westgard multi-rules. The laboratory participated in external quality assessments organized by the National Center for Clinical Laboratories and Fujian Neonatal Screening Program, achieving satisfactory results. Interpretation: Initial weak positives (TSH 9–20 μIU/mL) required repeat heel blood testing; initial positives (TSH ≥20 μIU/mL) or retest results ≥9 μIU/mL were referred for confirmatory testing.

### 3.3 Confirmatory procedures

**Figure 1:**
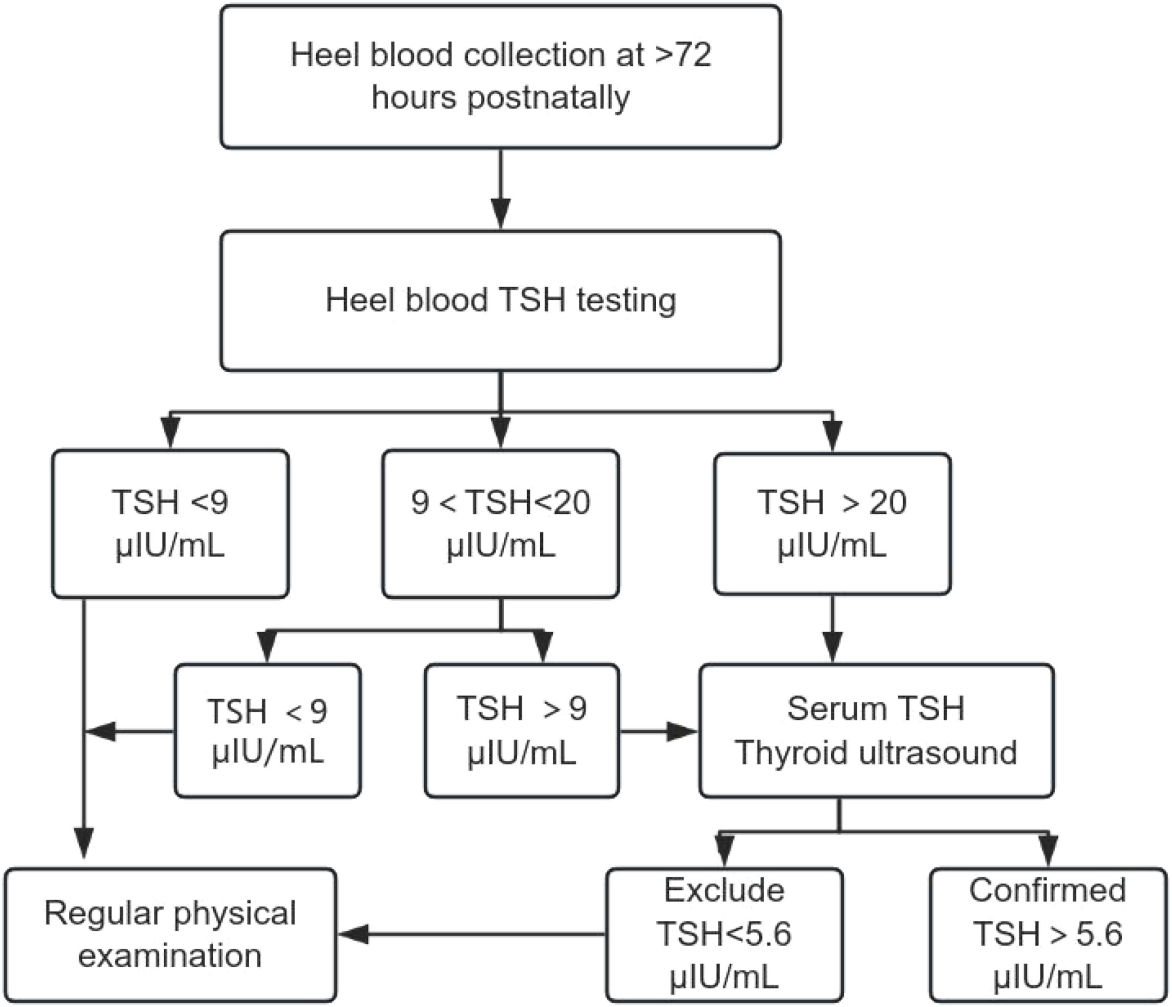
Confirmatory procedures.

Serological Testing: 2 mL of venous blood was collected, centrifuged, and analyzed for serum TSH, free triiodothyronine (FT3), and free thyroxine (FT4) using a Beckman DXI800 chemiluminescence immunoassay analyzer. Diagnostic Criteria: CH: Serum TSH >5.6 μIU/mL and FT4 <7.5 pmol/L; Hyperthyrotropinemia: TSH >5.6 μIU/mL and FT4 >7.5 pmol/L; Imaging: All suspected cases underwent thyroid ultrasound to assess developmental abnormalities (e.g., ectopic or absent thyroid). (**Figure 1**)

## 4 Statistical analysis

Data were analyzed using SPSS 20.0 and GraphPad Prism 9.0. TSH concentrations followed a positively skewed distribution, described by median (P50) and percentiles (P5, P95, P99). Optimal TSH cut-offs were determined via: Percentile Method: P99 as a potential threshold; ROC Curve Analysis: Using confirmed CH/hyperthyrotropinemia cases as the gold standard, area under the curve (AUC), sensitivity, and specificity were calculated. The Youden index maximum was selected as the optimal cut-off. Use the t-test to compare the differences in count data between the two groups, Statistical significance was set at P < 0.05.

## 5 Results

### 5.1 Overview of neonatal CH screening and TSH cut-off optimization

**Figure 2:**
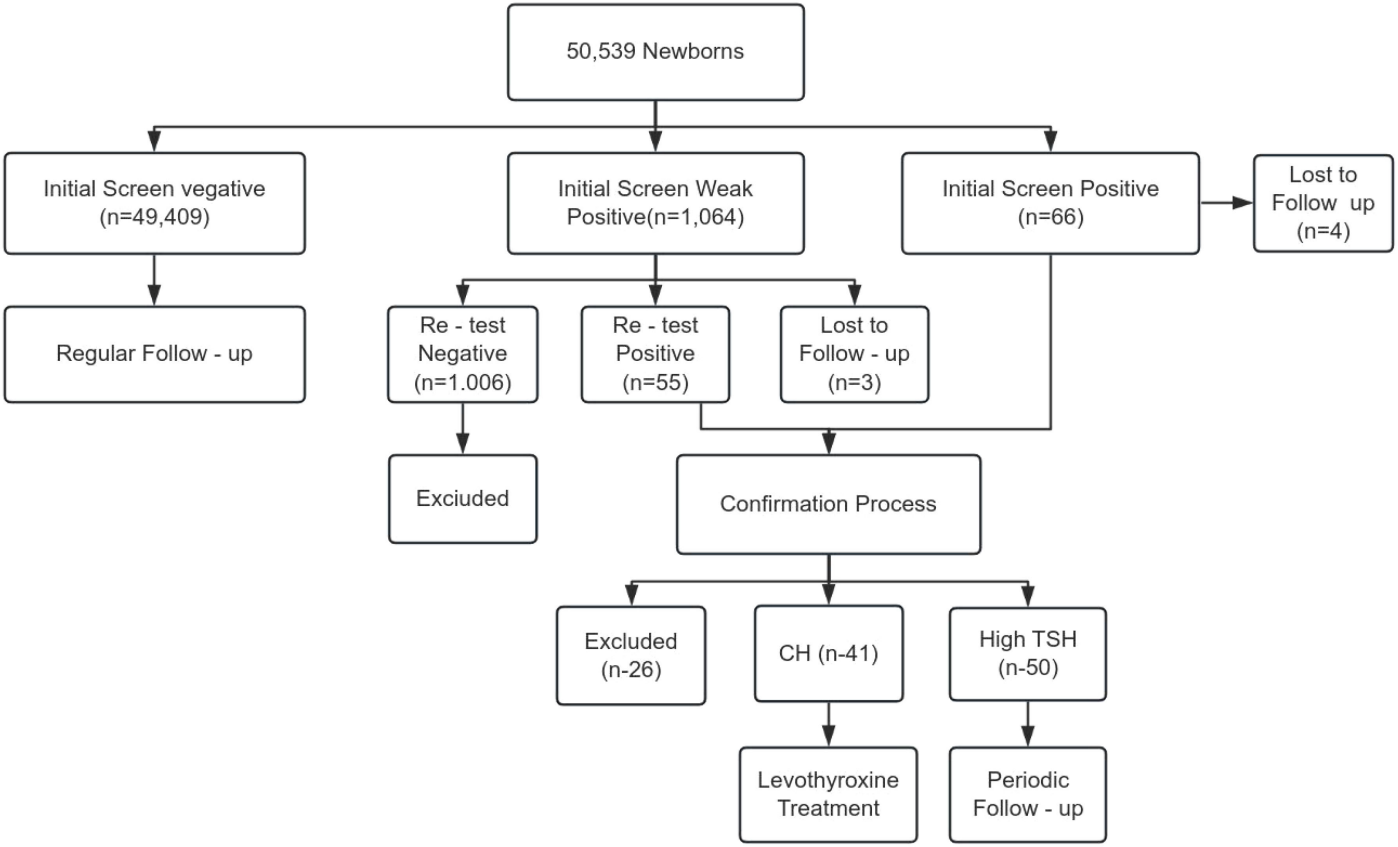
Overview of neonatal CH screening.

From July 2020 to November 2022, 50,539 newborns in Putian underwent CH screening. Initial screening identified 1,064 weak positives (TSH 9–20 μIU/mL) and 66 positives (TSH ≥20 μIU/mL), with 7 cases lost to follow-up. Confirmatory testing diagnosed 41 CH cases (Detection rate: 1:1,232) and 50 cases of hyperthyrotropinemia (Total detection rate: 1:555) (**Figure 2**).

### 5.2 Optimization and diagnostic performance of TSH cut-offs

**Figure 3:**
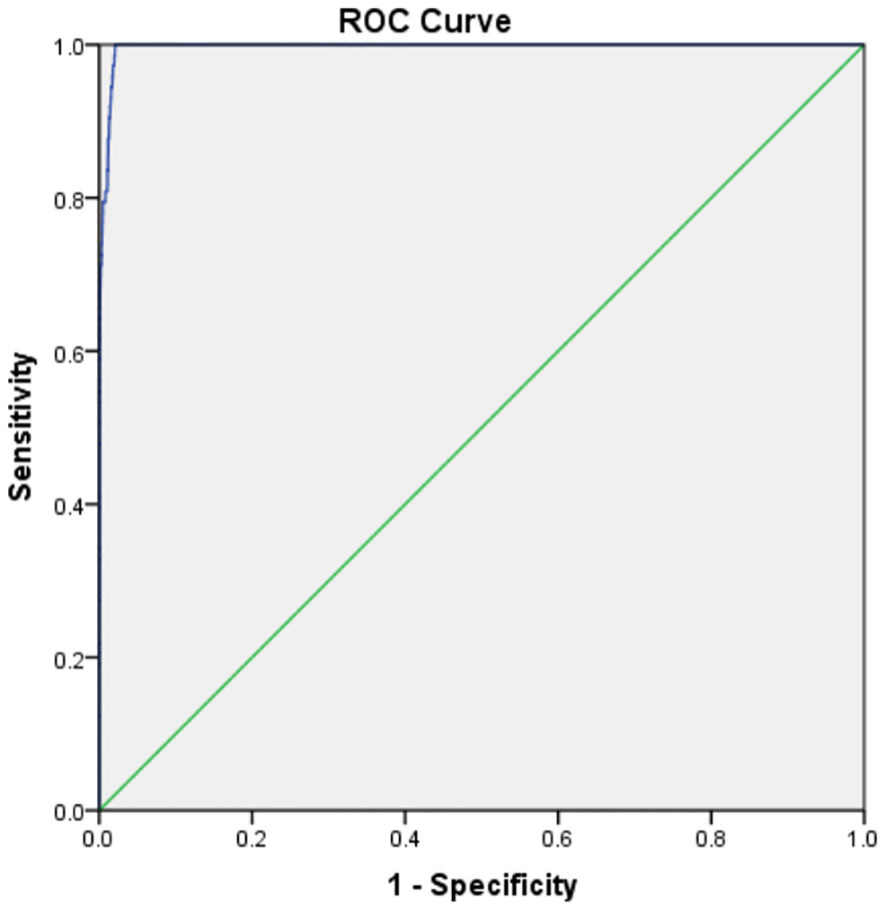
The ROC curve of TSH.

The P99 percentile method identified a TSH cut-off of 11.1 μIU/mL. ROC curve analysis revealed an optimal cut-off range of 9.33–9.43 μIU/mL (sensitivity: 100%, specificity: 98.1%, AUC = 0.997, P < 0.0001). A revised cut-off of 9.5 μIU/mL was adopted, reducing recall rates by 10.62% (avoiding 240 retests) but missing 1 hyperthyrotropinemia case (There were no children with missed diagnosis of CH) (**Figure 3**, **Table 1**).

**Table 1:** Diagnostic performance of TSH cutoffs.

(ROC Analysis)			
TSH Cutoff (μIU/mL)	Sensitivity (%)	Specificity (%)	Youden Index
8.95	1	0.021	0.979
9.025	1	0.021	0.979
9.275	1	0.02	0.98
9.325	1	0.019	0.981
9.375	1	0.019	0.981
9.425	1	0.019	0.981
9.475	0.988	0.018	0.97
9.525	0.976	0.018	0.958
9.575	0.976	0.018	0.958
9.625	0.976	0.017	0.959
9.675	0.965	0.017	0.948
9.775	0.965	0.016	0.949

### 5.3 Seasonal impact on screening results

**Figure 4:**
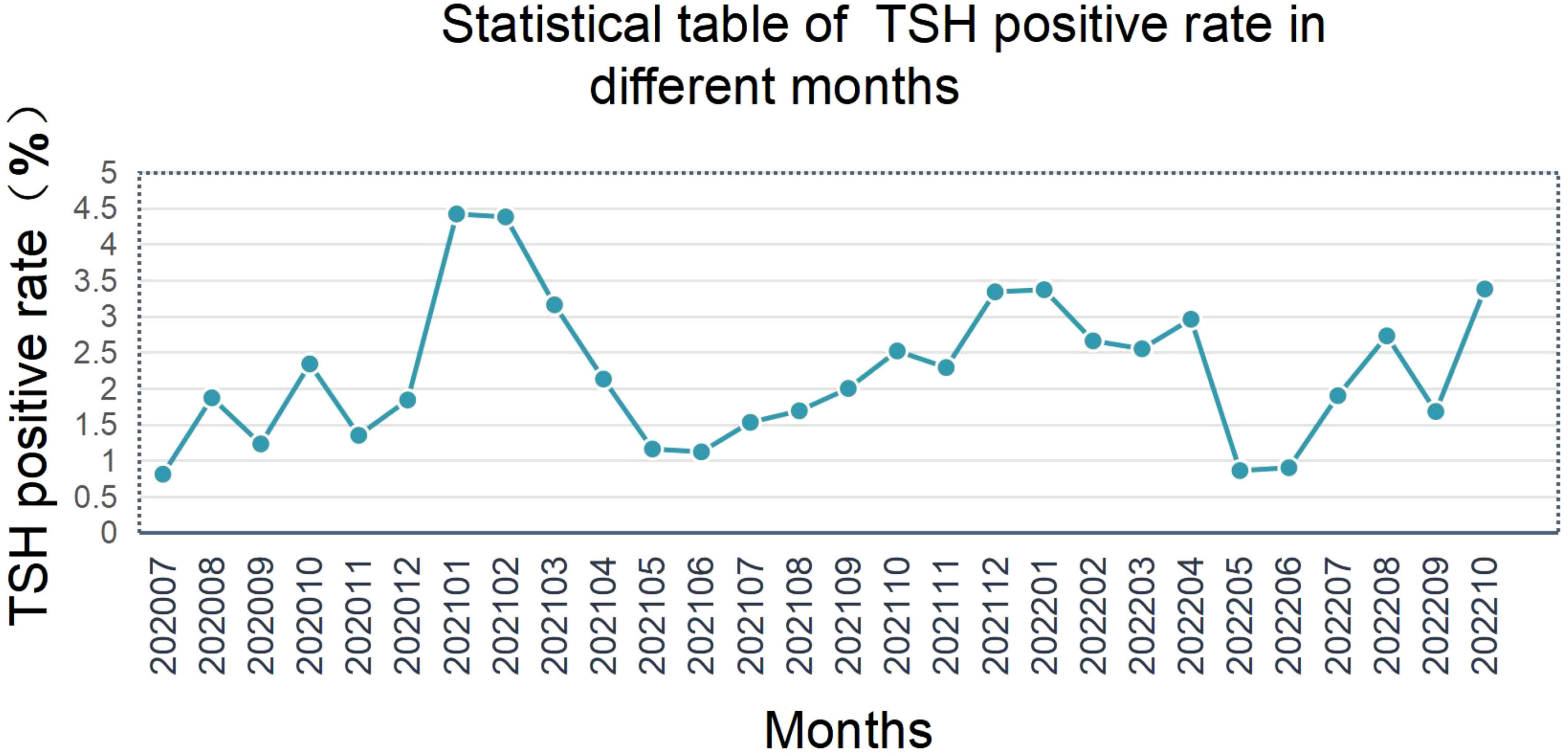
Statistical table of TSH positive rate in different months.

Initial TSH positivity exhibited significant seasonal variation, with winter (December–February) rates 32.5% higher than summer (June–August) (**Figure 4**). Lower ambient temperatures may stimulate neonatal metabolic activity, leading to physiological TSH elevation.

### 5.4 Analysis of the clinical characteristics of confirmed children

Our laboratory confirmed a total of 91 cases (41 CH patients and 50 cases of hyperthyrotropinemia). The clinical characteristics are shown in **Table 2**. Compared with normal newborns, the CH detection rate was 1.6 times higher in preterm infants and 2.3 times higher in low-birth-weight infants.

**Table 2:** Delivery characteristics of 91 confirmed children.

Group		Patient (n=91)	Normal (n=50448)	Rate (%)
Gender	male	52	27265	0.19
	female	39	23183	0.16
Gestational	Premature birth (<37 weeks)	8	2856	0.28
	Full-term (>37 weeks)	83	47592	0.17
Weight	<2500g	8	2013	0.39
	>2500g	83	48435	0.17

Among the 91 confirmed children, there were 41 children with CH and 50 children with hyperTSH syndrome.

Among the 91 cases, 83 (91.2%) had normal thyroid ultrasound findings, while 8 (8.7%) showed abnormalities (**Table 3**). Additionally, 18 (19.9%) had a family history of thyroid-related diseases.

**Table 3:** Clinical characteristics of 8 children with abnormal thyroid color Doppler ultrasound.

Number	Diagnosis	Gender	weight (g)	Gestational	dry blood spot, TSH	Serum TSH	Thyroid color Doppler ultrasound result	Family history
1	CH	female	2800	36^+2^	62.25	>48.9	Thyroid nodule	none
2	CH	male	3450	41^+1^	226	>48.9	Hypothyroidism	none
3	CH	male	3800	41^+2^	130.5	>48.9	Hypothyroidism	none
4	CH	female	3180	40^+2^	151	>48.9	Thyroid deficiency	hyperthyroidism in the mother
5	HyperSHemia	male	3800	40^+5^	9.4	19.84	Thyroid nodule	none
6	HyperSHemia	male	3550	39^+4^	35.8	>48.9	Hypothyroidism	Hypothyroidism in the mother
7	HyperSHemia	male	3250	40^+1^	105.05	>48.9	Hypothyroidism	Mother’s hyperthyroidism
8	HyperSHemia	female	2800	39^+1^	26.9	37.5	Ectopic thyroid	none

**Figure 5:**
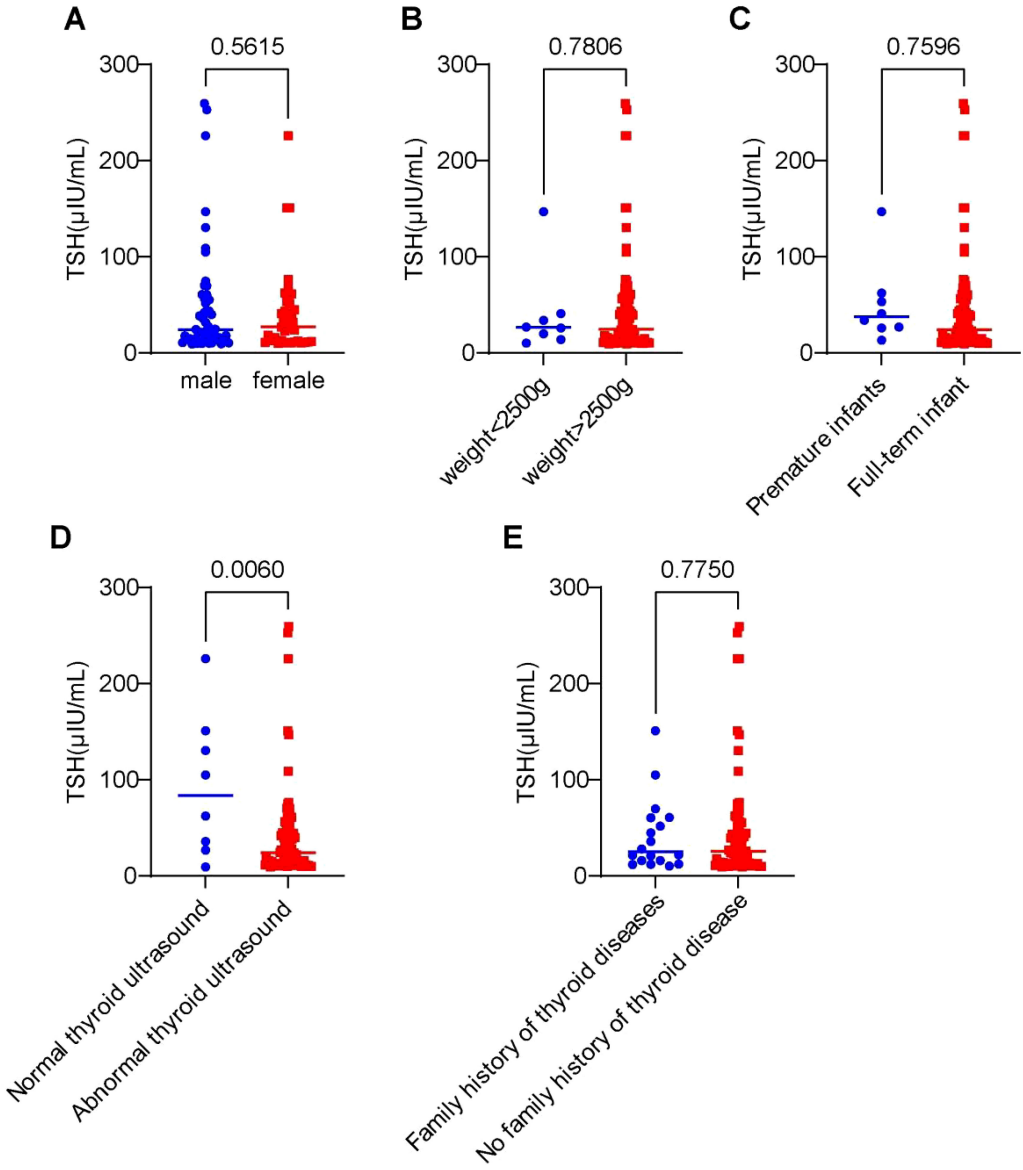
Differences in initial dried blood spot (TSH) screening levels among 91 cases stratified by clinical characteristics. **(A)** Impact of gender on TSH concentration levels. **(B)** Influence of birth weight on TSH expression levels. **(C)** Effect of gestational age on TSH concentration. **(D)** Association between thyroid structural abnormalities and TSH levels. **(E)** Role of family history of thyroid disorders in TSH expression.

**Figures 5A-E** presents the differences in initial dried blood spot (DBS) TSH screening levels based on: Gender、Birth weight、Gestational age、Thyroid ultrasound findings、Family history of thyroid disease.

Statistical analysis revealed: No significant differences in initial TSH levels between groups for sex, birth weight, gestational age, or family history (P > 0.05). Abnormal thyroid ultrasound findings (agenesis, dysplasia, ectopy, nodules) were associated with significantly higher initial heel blood TSH levels compared to normal ultrasound cases (P < 0.05).

### 5.5 Necessity of secondary screening in initially negative cases

A subset of neonates (preterm, low birth weight, or with comorbidities) may exhibit delayed TSH elevation. Through systematic review of outpatient records for missed cases, we identified 68 infants with initially negative dried blood spot (DBS) TSH screening results (median: 3.5 μIU/mL) who subsequently demonstrated thyroid dysfunction via serum TSH testing during hospitalization or follow-up visits. Among these, two cases were clinically diagnosed with congenital hypothyroidism (CH) (initial DBS TSH: 0.2 and 6.2 μIU/mL, respectively) and showed significant improvement in jaundice following levothyroxine treatment. The remaining 66 cases (median initial DBS TSH: 3.5 μIU/mL) were diagnosed with hyperthyrotropinemia and received phototherapy for jaundice with scheduled thyroid function monitoring. Notably, preterm infants accounted for 27.9% (19/68) of these missed cases, underscoring the necessity for secondary screening in this population. Concurrent hyperbilirubinemia was present in 57 cases (83.8%) (**Table 4**).

**Table 4:** Clinical characteristics of the 68 missed cases.

	Gestational		Birth weight		Clinical diagnosis		
	<37 weeks	37–42 weeks	<2500g	>500g	Hyperbilirubinemia	Preterm infant	Other
Case	19 (27.9%)	49 (72.1%)	12 (17.6%)	56(82.4%)	57 (83.8%)	5 (7.4%)	6 (8.8%)

## 6 Discussion

Since 2020, Fujian has implemented a free neonatal CH screening program, with Putian Maternal and Child Health Hospital serving as the regional screening center. By November 2022, 50,539 newborns had been screened. This study systematically evaluated the impact of TSH cut-off optimization on screening efficacy, highlighting the necessity of region-specific adjustments and providing critical insights for clinical practice. Preliminary data revealed a CH detected rate of 1:1,232 in Putian, higher than the national average (7), potentially attributable to coastal iodine nutrition patterns, increasing maternal age, and screening cut-off settings (8, 9).

### 6.1 Scientific and practical rationale for TSH cut-off optimization

ROC curve analysis identified an optimal TSH cut-off range of 9.33–9.43 μIU/mL (AUC = 0.997), aligning with Delgado et al.’s (2021) assertion that “regional cut-offs must balance sensitivity and cost-effectiveness” (4, 10). Adopting a revised threshold of 9.5 μIU/mL reduced recall rates by 10.62% (avoiding 240 retests) while missing only 1 hyperthyrotropinemia case (later confirmed as physiological TSH elevation through follow-up). This supports the international consensus that mild TSH elevation requires clinical monitoring rather than overtreatment (11, 12). Recent studies further validate that modest TSH adjustments can enhance screening efficiency by 20%–30% (13), emphasizing the need to balance diagnostic sensitivity with resource allocation.

### 6.2 Mechanistic insights into seasonal variability

The observed winter-summer disparity in TSH positivity (32.5% higher in winter; **Figure 4**) may stem from cold-induced metabolic activation and compensatory TSH elevation in newborns. McMahon et al. (2021) reported similar trends but found no seasonal correlation with CH diagnosis rates (6). A 2023 multicenter study noted that a 5°C temperature drop elevates neonatal TSH by 1.2 μIU/mL (14), corroborating our findings. Future protocols could incorporate temperature correction factors or seasonal cut-off adjustments to improve specificity.

### 6.3 Risk factor analysis for neonatal CH/hyperthyrotropinemia

The prevalence of mild CH has shown a steady increase in recent years (15–17). Mild CH may represent either permanent or transient thyroid dysfunction. Family history of thyroid disorders, thyroid ultrasonography, and genetic testing may help elucidate the etiology and determine the necessity for (long-term) treatment (18).

Our study demonstrated that neonates with abnormal thyroid ultrasound findings had significantly higher initial dried blood spot (DBS) TSH levels compared to those with normal ultrasound results (P < 0.05). These infants also exhibited relatively elevated serum TSH levels upon confirmation. Notably, 8 cases continue to receive levothyroxine supplementation to date, suggesting a higher likelihood of progressing to permanent CH. These findings indicate that thyroid ultrasonography can provide valuable guidance for CH diagnosis and management.

For most CH cases with normal thyroid ultrasound findings and no family history, low-dose levothyroxine therapy may suffice. A trial discontinuation of treatment can be considered from 6 months of age, with subsequent thyroid function monitoring to assess the need for further intervention (17).

Based on pathophysiology, CH has traditionally been classified into thyroid dysgenesis (TD) and dyshormonogenesis (DH), with genetic origins identified in some cases. Population genomic data (gnomAD v2.1.1) have revealed 12 autosomal recessive gene mutations (SLC5A5, TPO, TG, IYD, DUOXA2, DUOX2, TSHR, SLC26A7, GLIS3, FOXE1, TSHB, TRHR) that play critical roles in thyroid morphogenesis, hormonogenesis, and function. Among these, DUOX2 and DUOXA2 mutations exhibit the highest detection rates, followed by TG and TPO mutations (19), though their precise molecular mechanisms require further investigation.

Neonatal CH results from combined genetic and environmental factors, with 80%-85% of cases attributed to TD and the remainder to DH (20). In our cohort of 91 confirmed cases, 18 (19.9%) had a family history of thyroid disorders, including 1 case of thyroid agenesis and 2 cases of thyroid hypoplasia. This demonstrates that neonates born to mothers with a family history of thyroid disease have significantly higher CH risk than those without such history (21).

For families with a history of thyroid disorders, we recommend genetic mutation analysis to: Guide personalized treatment strategies、 Elucidate disease pathogenesis、 Predict recurrence risk and Assess future thyroid disease risk in family members (particularly siblings) (17, 22).

### 6.4 Imperative for secondary screening in high-risk groups

Neonatal jaundice is clinically categorized into physiological and pathological types. Physiological jaundice typically resolves spontaneously within the first postnatal week without therapeutic intervention and carries an excellent prognosis. In contrast, pathological jaundice demonstrates prolonged duration with complex etiologies. Without addressing underlying causes, phototherapy alone often leads to hyperbilirubinemia recurrence and treatment failure. Severe cases may progress to kernicterus, resulting in permanent neurological sequelae including cerebral palsy, intellectual disability, and seizures (23, 24).

Our study identified that 83.8% (57/68) of false-negative cases in initial heel prick screening presented with concurrent hyperbilirubinemia. This observation aligns with Tanaka et al.’s (2007) “bilirubin-thyroid axis interference hypothesis” (25), suggesting that neonatal hyperbilirubinemia may impair thyroid function, with disease severity correlating with the degree of thyroid hormone suppression. The proposed mechanism involves elevated serum bilirubin levels directly affecting thyroid homeostasis.

Contrasting with Tanaka’s findings, Cheng et al. postulated that reduced basal metabolic rate in CH infants leads to impaired hepatic maintenance of high-energy phosphate-bound bilirubin. Concurrently, decreased intestinal motility delays meconium excretion, enhancing enterohepatic bilirubin recirculation and consequent hyperbilirubinemia or prolonged jaundice (26). Tiker et al. reported 5 CH neonates presenting with severe hyperbilirubinemia within two postnatal weeks, after excluding common etiologies (ABO/Rh incompatibility, G6PD deficiency, etc.), suggesting hyperbilirubinemia as a potential early CH manifestation (27).

Our cohort included 2 hyperbilirubinemic CH cases where combined levothyroxine and phototherapy achieved significant bilirubin reduction. These findings underscore the necessity for thyroid function monitoring in hyperbilirubinemic neonates, even with initial negative dried blood spot TSH screening, to enable early intervention and improve neurodevelopmental outcomes.

Notably, our study revealed that preterm infants exhibited a 1.6-fold higher CH detection rate compared to term neonates, while low birth weight infants showed a 2.3-fold increased incidence versus the general population. Among the 68 missed cases in our cohort, preterm infants accounted for 27.9%. This phenomenon may be attributed to the immaturity of the hypothalamic-pituitary-thyroid (HPT) axis in these infants, leading to delayed TSH elevation. Notably, the degree of HPT axis immaturity appears inversely correlated with both gestational age and birth weight, resulting in more prolonged TSH elevation delays in more premature and smaller infants (28).

These findings align with the report by Xie et al., which identified that false-negative CH screening results predominantly occurred in preterm, low birth weight, and same-sex twin infants (29). In our study, the median initial dried blood spot (DBS) TSH level in the 68 missed cases was only 3.5 μIU/mL, suggesting that even with optimal cutoff value adjustments, cases with delayed TSH elevation may still evade detection. This underscores the critical need for implementing a secondary screening protocol.

Our data demonstrated that conducting secondary screening at 15–30 days of life for initially screen-negative infants with high-risk factors enabled the identification of an additional 32.3% (272/842) of CH cases. These results strongly support the 2024 updated guidelines from the European Society for Paediatric Endocrinology, which explicitly recommend secondary screening at 2–4 weeks of age for preterm infants, low birth weight neonates, and those with hyperbilirubinemia (30). The remarkable consistency between our findings and these international recommendations reinforces the clinical validity of our conclusions.

### 6.5 Innovations and limitations

This study pioneers a large-scale neonatal CH screening database for southeastern coastal China and proposes a comprehensive strategy integrating regional cut-offs, seasonal adjustments, and secondary screening for high-risk populations. However, limitations include: Incomplete 3-year neurodevelopmental assessments for 20% of CH cases, potentially affecting long-term outcome evaluations; Lack of systematic analysis on maternal iodine nutrition’s role in CH incidence, warranting future studies aligned with Fujian’s iodine intake profile (6, 31); Secondary screening protocols remain unimplemented in Putian’s public health framework, necessitating policy-driven initiatives (32).

### 6.6 Future directions

Building on 2025 neonatal screening trends, we recommend: Developing AI-driven TSH correction models to mitigate seasonal/environmental interference (33); Establishing multicenter networks to validate regional cut-offs across Fujian’s coastal cities; Investigating correlations between cord blood and dried blood spot TSH levels to expedite screening (34).

## 7 Conclusion

1. A TSH cutoff value of 9.5 μIU/mL is recommended for neonatal congenital hypothyroidism (CH) screening in Putian City to maintain high sensitivity while reducing unnecessary utilization of medical resources.
2. Thyroid ultrasound and genetic testing may facilitate etiological clarification, determination of long-term therapeutic necessity, and prediction of thyroid disorder risks in family members (particularly future siblings).
3. Integration of seasonal adjustments with secondary screening protocols for high-risk populations (e.g., prematurity, low birth weight, hyperbilirubinemia) may further enhance screening efficacy.

## Data Availability

The original contributions presented in the study are included in the article/supplementary material. Further inquiries can be directed to the corresponding author.

## References

[1] Brady J Cannupp A Myers J Jnah AJ. Congenital hypothyroidism. Neonatal Netw. (2021) 40:377–85. doi: 10.1891/11-T-699 34845088

[2] van Trotsenburg P Stoupa A Leger J Rohrer T Peters C Fugazzola L. Congenital hypothyroidism: A 2020–2021 consensus guidelines update. Thyroid. (2021) 31:387–419. doi: 10.1089/thy.2020.0333 33272083 PMC8001676

[3] Zhao Z Chen C Sun X Zhou D Huang X Dong H. Newborn screening for inherited metabolic diseases using tandem mass spectrometry in China: Outcome and cost-utility analysis. J Med Screen. (2022) 29(1):12–20. doi: 10.1177/09691413211021621 34102920

[4] Delgado JA Bauça JM Pérez Esteban G Caimari Jaume M Robles Bauza J. Challenges in screening for congenital hypothyroidism: Optimization of thyrotropin cut-off values. Clin Chim Acta. (2021) 512:20–5. 10.1016/j.cca.2020.11.009 33238181

[5] Lain S Trumpff C Grosse SD Olivieri A Van Vliet G. Are lower TSH cutoffs in neonatal screening for congenital hypothyroidism warranted? Eur J Endocrinol. (2017) 177(5):D1–D12. doi: 10.1530/EJE-17-0107 28694389 PMC5763485

[6] McMahon R DeMartino L Sowizral M Powers D Tracy M Caggana M. The Impact of Seasonal Changes on Thyroxine and Thyroid-Stimulating Hormone in Newborns. Int J Neonatal Screen. (2021) 7(1):8. doi: 10.3390/ijns7010008 33546274 PMC7930942

[7] Mehran L Khalili D Yarahmadi S Amouzegar A Mojarrad M Ajang N. Worldwide recall rate in newborn screening programs for congenital hypothyroidism. Int J Endocrinol Metab. (2017) 15(3):e55451. 29201074 10.5812/ijem.55451 PMC5702453

[8] Pal N Samanta SK Chakraborty A Chandra NK Chandra AK. Interrelationship between iodine nutritional status of lactating mothers and their absolutely breast-fed infants in coastaldistricts of Gangetic West Bengal in India. Eur J Pediatr. (2018) 177(1):39–45. doi: 10.1007/s00431-017-3025-6 29063209

[9] Harris KB Pass KA. Increase in congenital hypothyroidism in New York State and in the United States. Mol Genet Metab. (2007) 91(3):268–77. 10.1016/j.ymgme.2007.03.012 17512233

[10] Yang S Shi FT Leung PC Huang HF Fan J. Low Thyroid Hormone in Early Pregnancy Is Associated With an Increased Risk of Gestational Diabetes Mellitus. J Clin Endocrinol Metab. (2016) 101(11):4237–43. 10.1210/jc.2016-1506 27583471

[11] Oren A Wang MK Brnjac L Mahmud FH Palmert MR. Mild neonatal hyperthyrotrophinaemia: 10-year experience suggests the condition is increasingly common but often transient. Clin Endocrinol (Oxf). (2013) 79(6):832–7. doi: 10.1111/cen.12228 23611595

[12] Vigone MC Capalbo D Weber G Salerno M. Mild Hypothyroidism in Childhood: Who, When, and How Should Be Treated? J Endocr Soc. (2018) 2(9):1024–39. doi: 10.1210/js.2017-00471 PMC6117400 30187015

[13] Ye L Yin Y Chen M Gong N Peng Y Liu H. Combined genetic screening and traditional newborn screening to improve the screening efficiency of congenital hypothyroidism. Front Pediatr. (2023) 11:1185802. doi: 10.3389/fped.2023.1185802 37252044 PMC10213735

[14] Ho NT Busik JV Resau JH Paneth N Khoo SK. Effect of storage time on gene expression data acquired from unfrozen archived newborn blood spots. Mol Genet Metab. (2016) 119(3):207–13. doi: 10.1016/j.ymgme.2016.08.001 PMC5083152 27553879

[15] McGrath N Hawkes CP McDonnell CM Cody D O'Connell SM Mayne PD. Incidence of Congenital Hypothyroidism Over 37 Years in Ireland. Pediatrics. (2018) 142(4):e20181199. 30242075 10.1542/peds.2018-1199

[16] Zhou D Yang R Huang X Huang X Yang X Mao H. Results of neonatal screening for congenital hypothyroidism and hyperphenylalaninemia in Zhejiang province from 1999 to 2022. Zhejiang Da Xue Xue Bao Yi Xue Ban. (2023) 52(6):683–92. 10.3724/zdxbyxb-2023-0473 PMC10764193 38105685

[17] van Trotsenburg P Stoupa A Léger J Rohrer T Peters C Fugazzola L. Congenital hypothyroidism: a 2020-2021 consensus guidelines update-an endo-european reference network initiative endorsed by the european society for pediatric endocrinology and the european society for endocrinology. Thyroid. (2021) 31(3):387–419. doi: 10.1089/thy.2020.0333 33272083 PMC8001676

[18] Vigone MC Capalbo D Weber G Salerno M. Mild hypothyroidism in childhood: who, when, and how should be treated? J Endocr Soc. (2018) 2(9):1024–39. 10.1210/js.2017-00471 PMC6117400 30187015

[19] Park KS. Analysis of worldwide carrier frequency and predicted genetic prevalence of autosomal recessive congenital hypothyroidism based on a general population database. Genes (Basel). (2021) 12(6):863. doi: 10.3390/genes12060863 34200080 PMC8228807

[20] Nettore IC Cacace V De Fusco C Colao A Macchia PE. The molecular causes of thyroid dysgenesis: a systematic review. J Endocrinol Invest. (2013) 36(8):654–64. 10.3275/8973 23698639

[21] Ahn J Jeong H. Genetic etiology of permanent congenital hypothyroidism in korean patients: a whole-exome sequencing study. Int J Mol Sci. (2025) 26(9):4465. 40362701 10.3390/ijms26094465 PMC12072708

[22] Long W Guo F Yao R Wang Y Wang H Yu B. Genetic and phenotypic characteristics of congenital hypothyroidism in a chinese cohort. Front Endocrinol (Lausanne). (2021), 12:705773. doi: 10.3389/fendo.2021.705773 PMC8446595 34539567

[23] Gottimukkala SB Lobo L Gautham KS Bolisetty S Fiander M Schindler T. Intermittent phototherapy versus continuous phototherapy for neonatal jaundice. Cochrane Database Syst Rev. (2023) 3(3):CD008168. doi: 10.1002/14651858.CD008168.pub2 36867730 PMC9979775

[24] Par EJ Hughes CA DeRico P. Neonatal Hyperbilirubinemia: Evaluation and Treatment. Am Fam Physician. (2023) 107(5):525–34. 37192079

[25] Tanaka K Shimizu T Hosaka A Tokita A Shiga S Yamashiro Y. Serum free T4 and thyroid stimulating hormone levels in preterm infants and relationship between these levels and respiratory distress syndrome. Pediatr Int. (2007) 49(4):447–51. doi: 10.1111/j.1442-200X.2007.02390.x 17587266

[26] Cheng SW Chiu YW Weng YH. Etiological analyses of marked neonatal hyperbilirubinemia in a single institution in Taiwan. Chang Gung Med J. (2012) 35(2):148–54. 10.4103/2319-4170.106157 22537929

[27] Tiker F Gurakan B Tarcan A Kinik S. Congenital hypothyroidism and early severe hyperbilirubinemia. Clin Pediatr (Phila). (2003) 42(4):365–6. 10.1177/000992280304200411 12800733

[28] Kaluarachchi DC Allen DB Eickhoff JC Dawe SJ Baker MW. Thyroid-stimulating hormone reference ranges for preterm infants. Pediatrics. (2019) 144(2):e20190290. 31311840 10.1542/peds.2019-0290

[29] Xie T Tan M Jiang X Feng Y Chen Q Mei H. Clinical features and outcomes of 31 children with congenital hypothyroidism missed by neonatal screening. Zhejiang Da Xue Xue Bao Yi Xue Ban. (2022) 51(3):314–20. doi: 10.3724/zdxbyxb-2022-0213 PMC10251184 36207837

[30] Persani L Rodien P Moran C Visser WE Groeneweg S Peeters R. European Thyroid Association Guidelines on diagnosis and management of genetic disorders of thyroid hormone transport, metabolism and action. Eur Thyroid J. (20242024) 13(4):e240125. doi: 10.1530/ETJ-24-0125 38963712 PMC11301568

[31] Ji S Wu X Wu J Chen D Chen Z. Serum iodine concentration and its associations with thyroid function and dietary iodine in pregnant women in the southeast coast of China: a cross-sectional study. Front Endocrinol (Lausanne). (2023) 14:1289572. 38027098 10.3389/fendo.2023.1289572 PMC10665901

[32] Association NHCOTPROCSOOCM. National Health Commission guidelines for diagnosis and treatment of colorectal cancer 2023 in China (English version). Chin J Cancer Res. (2023) 35(3):197–232. doi: 10.21147/j.issn.1000-9604.2023.03.01 37440823 PMC10334494

[33] Delanghe JR Van Elslande J Godefroid M Speeckaert MM Maenhout TM. Interpretation of TSH results can be improved by reference values fluctuating in time. Horm Mol Biol Clin Investig. (2024) 46(1):21–6. doi: 10.1515/hmbci-2024-0043 39344474

[34] Guo X Suo F Wang Y Yu D Wang Y Dong B. Metabolite biomarkers of screening neonatal congenital hypothyroidism based on dried blood spot metabolomics. Anal Bioanal Chem. (2025) 417(13):2889–902. doi: 10.1007/s00216-025-05828-w 40094997

